# Compliance with radiation protection among radiographers in Eswatini public health facilities

**DOI:** 10.4102/hsag.v29i0.2727

**Published:** 2024-12-10

**Authors:** Amelia Shungube, Thandokuhle E. Khoza

**Affiliations:** 1 Department of Radiography, Faculty of Health Science, Durban University of Technology, Durban, South Africa

**Keywords:** radiation, radiation protection, radiation safety, radiation safety standards, ionising radiation, as low as reasonably achievable (ALARA), compliance

## Abstract

**Background:**

The consequence of non-compliance with patient radiation safety standards increases unnecessary radiation exposure with high chances of harmful biological effects. Radiographers are trained to prevent these harmful effects by enforcing radiation protection, which is achieved through proper techniques, equipment, shielding materials and beam collimation.

**Aim:**

The study aimed to explore compliance with radiation protection by radiographers in Eswatini public health facilities (PHFs).

**Setting:**

Eswatini PHFs with radiography departments (RDs) representing all four regions in the country.

**Methods:**

The study applied a qualitative exploratory design. Purposive sampling was used to select participants. In-depth face-to-face interviews were conducted with radiographers (who have at least 2 years of work experience) until data saturation was achieved with the 13th participant. Data were analysed thematically.

**Results:**

A total of three themes were identified from the data analysis, namely participants’ attitudes towards compliance with radiation safety standards; participants’ subjective norms; and perceived behavioural control factors.

**Conclusion:**

The study demonstrated radiographers’ awareness and knowledge of patient radiation safety standards. However, compliance with the standards remained a personal decision as radiographers are not obliged to comply. Moreover, defective lead protective devices, the unavailability of the full scope of lead protective designs, the inappropriate design of the RD building and unauthorised staff making unjustified X-ray requests contributed to non-compliance.

**Contribution:**

The study highlighted a gap in compliance with patient radiation safety standards that requires attention from Eswatini’s Ministry of Health (MOH).

## Introduction

Chronic radiation exposure can negatively impact every system in the body, causing health issues such as prenatal malformations, cancer, benign tumours and genetic disorders (Allam, Algany & Khider 2024:2). Moreover, radiation sickness (bleeding, anaemia, loss of bodily fluids and bacterial infection) is one of the more severe abnormalities (Allam et al. 2024:2; Lewis, Downing & Hayre 2022b). Radiographers who perform radiographic procedures are trained to use the least amount of necessary radiation (Health Physics Society 2021:1). Radiographers play a significant role in and are considered important to performing radiological examinations and supporting radiation exposure, thus their practice should always be optimised according to the ‘as low as reasonably achievable’ (ALARA) principle. Producing high-quality images while keeping patients’ doses as low as possible can be challenging; hence, radiographers need to ensure total compliance with radiation safety standards (Abuzaid et al. 2019:447; Ridzwani Mohd, Fritschi & Bhoo-Pathy 2023:459).

All radiographers must use the lowest exposure to obtain a diagnostic quality image and use specific techniques that minimise the patients’ risks associated with ionising radiation exposure. These techniques include using compression during examinations of the pelvic region and lumbar spine, using a gonad shield and asking women if they are pregnant (Christensen et al. 2024:1). However, some staff do not use these techniques consistently. Increasing compliance requires determining why staff are non-compliant. Therefore, this study aims to qualitatively explore why radiographers do not use these techniques (Christensen et al. 2024:1). The International Atomic Energy Agency (IAEA) works to prevent patients from being exposed to excessive and unintended radiation, while ensuring that radiation doses are commensurate with medical purposes (IAEA n.d.a. par. 4, line 1). These unintended exposures can result from an unsafe design or inappropriate use of medical radiation technology (IAEA n.d.a. par. 4, line 1). The IAEA intends to accomplish better safety standards by implementing radiation protection programmes and activities designed to enhance radiation safety conformance.

Ridzwani et al. (2023:459) conducted a study in Malaysia, concluding that radiographers showed poor adherence to radiation monitoring. The main reasons for the non-use of radioprotective garments were inadequate items and the need to prioritise other radioprotective garments. This was consistent with a study in hospitals affiliated with Mashhad University of Medical Sciences; Salmanvandi et al. (2015:1) reported that some personal shields and radioprotective garments have defects (tears, holes and cracks) and that 13% of them were unacceptable in terms of equivalent lead thickness (ELT) and needed to be replaced. Failure to replace them hindered radiographers’ efforts to comply with radiation safety. In addition, Lewis, Downing and Hayre (2022a:1) conducted research in South Africa and concluded that even though participants’ knowledge of radiation safety matched the mandated guidelines, limited internalisation of the knowledge made compliance a matter of personal choice. This was consistent with the findings of this study in Eswatini, as well as a study performed in Agra City (Sarman & Che Hassan 2016:433). Sarman and Che Hassan (2016) study concluded that only one of 31 radiographers complied with radiation safety, although they were all aware of radiation protection.

Furthermore, a South African study by Lewis et al. (2022a:1) found that radiographers reflected on their casual attitude (not very concerned with compliance with radiation protection) and noticed a similar attitude among their peers. The study showed this cavalier attitude becoming the norm. Patient and work-related impediments, such as, (1) being rushed during the imaging of trauma patients and challenging patients; (2) patient knowledge of radiation safety; (3) resources; (4) imaging referrals; and (5) inadequate training when transitioning from analogue to digital radiography, were identified as contributing to radiation safety non-compliance. To promote compliance, strategies such as additional education, research and a mentality shift were suggested (Lewis et al. 2022a:1).

Consistent with Lewis et al. (2022a:1), the study conducted by Eze et al. (2013:1) revealed that Lagos Metropolis radiographers’ knowledge of radiation safety was high but their adherence was low. The public health facilities (PHFs) in the study lacked most modern radiation safety equipment and accessories required improvement. The majority of X-ray machines were outdated, and quality assurance evaluations performed on these machines required improvement. Thus, it was recommended that radiographers in Lagos, Nigeria, should embrace current trends in radiation protection and make more concerted efforts to apply their knowledge in protecting patients from the harmful effects of ionising radiation (Eze et al. 2013:1).

Partap et al.’s (2019:1) study in Trinidad concluded that the level of knowledge among radiographers across the country was minimal. In addition, a global meta-analysis of four published studies obtained from search engines reviewed from the year 2009 to 2016, concluded that only one radiographer (12.9%) complied with radiation protection (Sarman & Hassan 2016:1). Similarly, a study conducted among radiographers in the central region of Ghana found that radiation protection practices were generally well-known and that radiation safety compliance was satisfactory (Fiagbedzi et al. 2022:1). This was, however, insufficient; they knew and had satisfactory compliance with radiation protection but there was still room for improvement to ensure that knowledge is applied to enhance appropriate safety measures, guarantee effective work and reduce the negative effects of ionising radiation (Fiagbedzi et al. 2022:1).

Although radiation protection is taught in the tertiary curriculum for radiography and there are international guidelines and policies for patient radiation protection, this study purports that there is still a need for continuous education and training for Eswatini radiographers (Dlamini & Kekana 2021:1). Furthermore, based on the researcher’s observation, there is a need for a greater understanding of their views and experiences regarding complying with radiation protection. Considering that the reasons for Eswatini radiographers’ radiation protection behaviour remains largely unexplored, there was a need for qualitative research as a starting point for addressing the complexities of radiation safety compliance issues in Eswatini’s PHFs. In addition, there was a lack of data on these issues that might shed light on the compliance with radiation protection among radiographers in Eswatini PHFs. This study, therefore, aimed to explore compliance with radiation protection among radiographers in Eswatini PHFs.

The theory of planned behaviour (TPB) posits that behaviour depends mostly on the intention to perform a behaviour. In this manner, the TPB shaped the theoretical framework of this study. Intention to perform a behaviour depends on three factors: attitude towards a behaviour, subjective norm and perceived behavioural control (Lewis et al. 2022b:48). Attitude towards a behaviour depends on behavioural beliefs and the subjective likelihood of the result of that behaviour. When assessing the result of a behaviour, an individual who accepts the result to be positive and places more noteworthy significance on the result will most likely proposed to perform the behaviour. Subjective norm is based on normative beliefs. Normative beliefs are an individual’s conviction that society, either a person or a bunch that is seen as being critical, accepts they ought to or ought not to perform a behaviour, known as social pressure (Lam et al. 2015:740; Lewis et al. 2022b:48). Perceived behavioural control is based on control beliefs and influences both intention and conduct. It takes into account the potential limitations of the activity as seen by the person and their confidence in performing the conduct (Hundah 2019:20; Lewis et al. 2022b:48). This study was guided by the constructivism paradigm, which implies understanding and clarifying what one knows, as well as deciding what sorts of information is conceivable, and how to guarantee that they are both satisfactory and genuine. The researcher conducted in-depth interviews with the radiographers in their departments in order to get their information and guarantee its authenticity (Ahmed 2008:3). The researcher thus explored the radiographers compliance with radiation protection in Eswatini PHFs utilising the TPB.

### Aim

The study aimed to explore radiographers’ compliance with radiation protection in Eswatini PHFs. The study also sought to make recommendations to Eswatini’s Ministry of Health (MOH) regarding appropriate compliance standards.

## Research methods and design

### Research design

Research design is defined as a framework of methods and techniques chosen by a researcher to combine various components of research logically so that the research problem is efficiently handled, providing insights into ‘how’ to conduct the research using a particular methodology. Thus, this study aimed to explore compliance with radiation safety standards among radiographers employed at Eswatini PHFs in an exploratory, qualitative manner. Furthermore, every researcher has a list of research questions that need to be assessed, which can be done through the research design (Khanday & Khanam 2023:367). As such, the sketch of how research should be conducted can be prepared using the research design (Khanday & Khanam 2023:367). This study was guided by the constructivist paradigm. The study was conducted in Eswatini PHFs with radiography departments (RDs) from all four regions in the country, namely Lubombo, Manzini, Shiselweni and Hhohho.

### Population and sampling

The study population included employed radiographers with work experience of at least 2 years at PHFs. The total population consisted of 45 radiographers, and 13 participants formed part of the sample population where data saturation was reached. The sampling technique used for this study was purposive. Purposive sampling or judgemental sampling selects the sample members solely based on the researcher’s expertise and judgement (Akpan & Piate 2023:65). When choosing a sample using purposive sampling, the researchers carefully select each person who will be a part of the sample (Akpan & Piate 2023:65). Purposive sampling is most effective in situations where there are only a restricted number of people in a population who possess qualities that a researcher expects from the target population (Akpan & Piate 2023:65).

### Participants’ recruitment

The recruitment procedure includes identifying potential research participants and providing them with information to determine their willingness to participate in a proposed study. In addition, the recruitment and retention of study participants are crucial to the overall success of the research study (Manohar et al. 2018:2). As rapport existed between the researcher and the participants, word-of-mouth was used to recruit interviewees; the sampling method was, therefore, solely purposive. In addition, after obtaining permission from the PHFs’ senior medical officers, the researcher communicated directly with the radiographers to request their participation in the study. An informational letter explaining the purpose of the study and ethical considerations was hand-delivered to the participants, who were then required to sign written consent before the commencement of the interview. Bias was reduced by not allowing the participants to read the interview questions before the interview onset and the researcher used a similar interview questions for all the participants and remained professional when conducting the interviews. Those interested indicated when they were available. All of them preferred meeting during work hours in a private room when the patient flow was slow within their respective RD.

### Data collection process and instrumentation

An in-depth face-to-face interview was conducted using a semi-structured interview guide as the data collection instrument. The interview guide was created in a way that ensured the questions are focussed, relevant, clear, concise and unbiased. They were aligned with the research objective, scope and main aspects of the topic. The open-ended questions were made in a way that allowed participants to share their opinions and experiences. Follow up probing questions to explore deeper insights and clarifications were used where needed. Data saturation was achieved at the 13th participant. Data saturation is defined as a point in the data collection process where study categories and themes become repetitive and redundant, and no new information regarding the research purpose emerges (Braun & Clarke 2019:4; Fusch & Ness 2019:1408).

The following nine key questions guided the interview process. If further clarifications were needed, subsidiary questions were used to probe:

- What is your understanding of patient radiation protection?
- How often do you observe patient radiation safety with your patients and caregivers in the room during radiation exposure? Always? Never? It depends. Explain.
- Describe your attitude towards radiation protection. Why?
- What measures do you employ to protect patients from unnecessary radiation exposure?
- How would you describe the compliance with radiation protection at this facility?
- What challenges/obstacles do you face that hinder you in applying radiation protection?
- What can be done to improve or encourage compliance with radiation protection in this facility?
- What radiation protection gears are available in the RD? Describe their condition.
- What can be done to foster compliance with radiation protection in this facility?

The 13 interviews were recorded using an audio recorder with the participants’ permission, and the researcher took notes throughout the interview. The interviews lasted approximately 30 min each and were conducted in a private, silent room within the RD, which was suitable for the participants.

### Data analysis

In qualitative data analysis, preparing the data entails transcribing text from interviews into word-processing files for analysis (Creswell & Cresswell 2018:308–309). All interview transcripts were transcribed verbatim by the researcher. Thematic data analysis was utilised as it is a robust, yet adaptable, method for analysing qualitative data that can be applied within various paradigmatic or epistemological orientations (Kiger & Varpio 2020:1). This methodology was appropriate for the study because it permitted the successful analysis of radiographers’ radiation protection compliance-related experiences, beliefs and behaviour (Kiger & Varpio 2020:1).

The researcher undertook data transcription directly after data collection. Repetitive listening to the audio recordings was performed to avoid missing important components related to the study (Asif & Rodrigues 2015:281). This research utilised Creswell and Cresswell’s (2018:308–309) six-step data analysis procedure as follows:

- The initial phase was to organise and prepare the data for analysis by transcribing and printing the interview transcripts.
- The second phase comprised of reading or examining all the data. In this study, the researcher read and comprehended the transcriptions after gaining a general understanding of the information and an opportunity to ruminate on its overall significance through the preceding steps.
- The third stage entailed coding all the data, organising the data by chunks (or text or image segments) and writing a category-representing word in the margins.
- The fourth phase involved the coding process to generate a description of the sub-themes and themes for analysis. The researcher intended to categorise the classification of the data based on the theoretical framework of the study following the coding process.
- The fifth step was to refine the presentation of the description and themes in the qualitative narrative.
- The final phase involved the interpretation of qualitative research findings.

### Trustworthiness

The qualitative study must be conducted systematically and methodologically to produce meaningful and useful results. To be considered credible, Nowel et al. (2017:1) advise that qualitative researchers must demonstrate that the data analysis was conducted in a precise, consistent and exhaustive manner by disclosing the methods of analysis in sufficient detail for the reader to determine whether the process is credible. Lincoln and Guba’s (1985) four criteria (credibility, transferability, dependability and conformity) were used to evaluate the study’s reliability.

Credibility was achieved through prolonged engagement, reflexivity and triangulation of interview data with the researcher’s notes and literature as a way to provide multiple perspectives (Creswell & Creswell 2018:315). To maintain confirmability, the researcher ensured that the findings were based on the participant’s exact responses and not on any potential researcher bias or personal motivations (Ahmed 2024:3–4). The co-author also reviewed the data scripts and consensus was reached about the findings, interpretations, conclusions and recommendations of the data. Transferability pertains to the degree to which the research findings can be extrapolated to alternative contexts or situations, populations or phenomena (Ahmed 2024:3). To demonstrate that the findings of this study can be applied to other contexts, circumstances and situations, the researcher ensured that all relevant information, including a detailed description of the research setting and research methods confirming the authenticity and validity of the study, was described in detail from the study’s context to its conclusion, thus allowing further research to be based on the findings (Ahmed 2024:3). Dependability was ensured by keeping an audit trail and by providing a detailed description of data gathering, analysis and interpretation (Ahmed 2024:2).

### Ethical considerations

The study was conducted following the Helsinki Declaration as revised in 2013. The study was approved by the Durban University of Technology Institutional Research Ethics Committee (DUT) (IREC 308/22). Further approval was obtained from the Eswatini Health and Human Research Review Board with reference number (EHHRRB 035/22) and the senior medical officers of each PHF. The participants were given information letters explaining the study’s purpose, and those interested in participating in this study signed the informed consent voluntarily, without being coerced before conducting the study. Confidentiality and privacy were also adhered to by keeping participants’ names private (e.g., Participant 1) and using codes such as HC1 for the PHFs during the data analysis process, and all transcripts (including audio recordings) are kept on a computer, which will be password-protected in a locked office for 5 years.

## Results

### Demographics of the participants

Participants were employed as diagnostic radiographers from PHFs in all four regions of Eswatini (Lubombo, Manzini, Shiselweni and Hhohho). Participants’ age ranged from 20 to 60 years. Most of them had between 2 and 5 years of experience while some had 15 years of experience. All four regions of the country were represented through PHFs with RDs. Of the total 13 participants, 4 were from Hhohho, 2 from Manzini, 4 from Shiselweni and 3 from Lubombo. The ratio of male to female participants was almost identical, with one female participant outnumbering males (Table 1).

**TABLE 1:** Demographics of participants.

Participant	Health facility	Gender	Age (in years)	Work experience (in years)	Region of health facility
1	H3	F	20–29	2–5	Shiselweni
2	H3	M	30–49	2–5	Shiselweni
3	HC4	M	20–29	2–5	Shiselweni
4	HC4	M	20–29	2–5	Shiselweni
5	HC2	F	30–49	2–5	Hhohho
6	HC2	M	30–49	2–5	Hhohho
7	H2	F	30–49	5–10	Manzini
8	HC3	F	30–49	2–5	Manzini
9	H5	F	30–49	2–5	Lubombo
10	H5	M	50–59	> 15	Lubombo
11	H4	F	30–49	5–10	Hhohho
12	HC1	M	30–49	5–10	Lubombo
13	H6	F	30–49	5–10	Lubombo

The main themes and sub-themes in this study (Table 2) were generated from the TPB. This theory explains that individual behaviour is influenced by behavioural intentions, which are a function of three main determinants, namely an individual’s attitude towards behaviour, subjective norms and perceived behavioural control (Nioi et al. 2018:3). Individual attitude towards behaviour refers to the extent to which a person evaluates the behaviour of interest favourably or negatively (LaMorte 2020:para 3, line 1). A subjective norm is the belief that an important person or group will approve or support a particular behaviour (Lam et al. 2015:740). Perceived behavioural control is an individual’s perceived ease of achieving a specific behaviour based on experience, which is reflected in the individual’s resources to achieve the behaviour (Li et al. 2023:4). Thus, these findings were developed from the existing TPB (Bingham 2023:2–4).

**TABLE 2:** Summary of themes arranged in line with the theory of planned behaviour.

Themes	Sub-Themes
1 Behavioural attitude towards compliance with radiation protection	1.1 Participants’ favourable attitude 1.2 Participants’ unfavourable attitude
2 Subjective norm of participants	2.1 Social factor/culture
3 Factors influencing participants’ perceived behavioural control	3.1 A lack of resources 3.2 Patient-related factors 3.3 Poor infrastructure 3.4 Unauthorised personnel requesting X-ray examination requests 3.5 Methods to encourage compliance

#### Theme 1: Behavioural attitude towards compliance with radiation protection

This theme comprised of two sub-themes related to (1) participants’ favourable attitudes towards radiation protection and (2) their unfavourable attitudes towards radiation protection.

*Sub-theme 1.1: Participant’s favourable attitudes:* In the interviews, most participants provided positive feedback regarding the radiographers’ experiences and knowledge of radiation protection in practice. Their responses included:

‘… I think radiation protection is about measures that have to be put in place to protect patients, colleagues, yourself, and the public from radiation exposure since radiation is dangerous, it can cause cancer and skin reddening to count a few so we are trying to minimise the exposure with all measures we can use like avoiding doing unnecessary exams to the patient, minimising repeats through explaining the procedure and demonstrate to the patient, collimate to the area of interest, using correct source to image distance [*SID*] for that procedure, lead apron to avoid exposing unnecessary structures and kVp [*high kilovoltage peak*] technique where necessary.’ (Participant 3, HC4, M, 20–29)

‘… I think radiation protection is basically about taking measures to protect the environment, the people around you, and yourself as a radiographer from radiation exposure, basically mostly use lead aprons depending on the procedure requested, using the ALARA principle minimising time, shield and using the required distance and also ensure caregivers are asked to go outside the room or given a lead apron to wear and be told to be away from the primary beam, failure may lead to stochastic and deterministic effects.’ (Participant 7, H2, F, 30–49)

*Sub-theme 1.2: Participants’ unfavourable attitude:* This study demonstrated that compliance with radiation protection was a personal matter or decision. Participants were not obliged and the use of radiation protection depended on the radiographer attending to the patient. Participants admitted that they had a negative attitude and slight negligence towards radiation protection as they believed that the patient’s radiation exposure was insufficient in causing any biological or genetic effects:

‘… it’s unlike that time when I was a student, now I use radiation protection as I want, no need to worry about anything or anyone like marks and supervisors, there is no one supervising me to check or any radiation safety officers [*RSO*] that can randomly visit to see if I am using the radiation protection measures I work on my own so I use them the way I want.’ (Participant 1, H3, F, 20–29)

‘… for me, I feel like the radiation that we are getting is small, we have that one apron there, and the other one is still in the box and I don’t want to lie to you, but sometimes I open the door when exposing while patients queue by the door, I open because it’s very hot in this room, but I know that radiation protection is something that I need to do.’ (Participant 5, HC2, F, 30–49)

‘We always protect kids because they still have a long time to live and protect pregnant women with a lead shield [*that’s*] our main focus … it is upon ourselves to take the initiative to protect our patients from radiation and currently we do not have any policies that obligate us to use radiation protection.’ (Participant 7, H2, F, 30–49)

#### Theme 2: Subjective norm of participants

This theme only produced one sub-theme. It related to the effects of social factors and cultures on participants’ use of protective equipment.

*Sub-theme 2.1: Social factors/culture:* The results of this study showed that there was no obligation or any policy or law that would force radiographers to comply with radiation safety standards in Eswatini. Hence, compliance with radiation protection was a personal choice, and their current routine behaviour was poor because it was conditional. Interestingly, participants showed increased conformance with radiation protection for children and pregnant women. This was influenced by the practices and ethos of their departmental colleagues:

‘Whether I use radiation or not it’s my issue or choice, I use radiation protection but it’s not always because we are a busy institution most of the time, we are rushing trying to push the queue we don’t have time.’ (Participant 7, H2, F, 30–49)

‘We don’t always use radiation protection because we are a busy institution we don’t have time even though this cannot be an excuse for pregnant women who are involved in accidents we do use radiation protection, and for kids from 0 to 2 years we always protect and we mainly focus on lead shielding, for those pregnant women we ensure that we explain the risk of the examination and do it only if the risks outweigh the benefits like with pregnant women in first trimester we avoid by all means to examine unless it’s a very critical patient, then we have got no other way.’ (Participant 9, H5, F, 30–49)

#### Theme 3: Factors influencing participants’ perceived behavioural control

Perceived behavioural control takes into account the potential constraints on the action as perceived by the individual and their confidence in performing the behaviour. This theme generated five sub-themes related to several factors that influenced participants’ compliance with radiation protection.

*Sub-theme 3.1: A lack of resources:* Participants explained that they often feel discouraged by the current poor condition of lead aprons. Participants further believed these are ineffective as no replacements have ever been provided, although they are desperately needed. Therefore, if allowed to ameliorate the situation, they would replace all existing aprons with new ones. These non-functional lead aprons also create challenges for radiographers who must assist expectant women. Participants expressed their comprehension through the following statements:

‘In some instances where lead aprons are available, one will find that they do not fulfil all roles, as lead devices are distinct and serve various purposes. Examples include waist aprons, thyroid shields, and lead gloves used during special procedures other than the common lead apron. Buy all the necessary equipment for shielding because we end up having to fold the apron for pregnant women which damages it because we don’t have wraparound, we only have lead aprons and they are not useful when doing exams like chest there are no way you can protect the patient with it otherwise it will get folded damaging it.’ (Participant 3, HC4, M, 20–29)

‘… buy all the necessary equipment for shielding.’ (Participant 11, H4, F, 30–49)

‘We end up having to fold the apron for pregnant women which damages it because we don’t have wrap-around.’ (Participant 3, HC4, M, 20–29)

‘… we only have lead aprons and they are not useful when doing exams like chest there is no way you can protect the patient with it otherwise it will get folded damaging it.’ (Participant 1, H3, F, 20–29)

‘… we are not sure of the equipment situation as far as radiation protection is concerned … provide RSOs to come now and then and see the challenges we face and work on it, including the equipment moreover, they can also do QA assessments for the rooms and the lead aprons if they are effective, we need radiation protection officers such people are needed so that you can improve or see if there are any discrepancies on the equipment and with us radiation workers.’ (Participant 1, H3, F, 20–29)

*Sub-theme 3.2: Patient-related factors:* Participants shared their perspectives on patient-related factors that prevent them from complying with radiation protection measures. These included long queues and patients complaining that the lead aprons were heavy. Participants stated:

‘Yes, we do face several challenges. Some of our patients complain that lead devices are too heavy and there is always a long queue it takes more time to observe the radiation safety measures.’ (Participant 7, H2, F, 30–49)

‘… for patients in intensive care unit [*ICU*], we always have no way to protect the adjacent patient because the bed is fixed mostly and there is no way to move it….’ (Participant 7, H2, F, 30–49)

‘Patients complain that the apron is heavy and I get forced not to use it even if I want to use what we have.’ (Participant 9, H5, F, 30–49)

*Sub-theme 3.3: Poor infrastructure:* Several participants expressed a lack of motivation to conform to radiation protection measures because of their departments’ poor infrastructure. This infrastructure may either be in the wrong location and/or the lack of structural radiation protection features for patients waiting outside or colleagues in adjacent departments. The walls were not lined with lead, and the RD was located between busy departments such as the laboratory, causing the patients and staff in other rooms to be exposed. The participants expressed their apprehension as follows:

‘The room walls are not shielded, I once placed a cassette for 3 days in the other room department and processed it later only to find it is exposed meaning patients and staff in the other room are being exposed unnecessarily and the patients on the queue are also receiving radiation exposure since the walls do not have lead, we are supposed to have a radiology department away from here because the infrastructure is not proper.’ (Participant 6, HC2, M, 30–49)

‘We are supposed to have a radiology department away from here because the infrastructure is not proper.’ (Participant 8, HC3, F, 30–49)

‘In our case, the main challenge we have is that we do not have a leaded door or any door at all but we have a curtain as the door. So that means all those who don’t know and pass by during exposure get exposed, that can be patients or any other staff.’ (Participant 7, H2, F, 30–49)

*Sub-theme 3.4: Unauthorised personnel requesting X-ray examination requests:* Participants raised the issue of unauthorised personnel (nurses) making X-ray requests. While there were no known rules and regulations forbidding nurses from requesting X-rays, the participants strongly believed that only physicians should be able to order X-ray procedures. This is because participants often receive unnecessary orders from nurses, such as for a patient who may have a minor skin abrasion but is sent in for an X-ray to rule out fractures. Therefore, they believed it is essential to educate employees about the hazards of radiation and radiation protection procedures. Participants based on their experiences stated:

‘… we find that we have to expose patients for unnecessary orders to rule out fracture while it’s clear there is no need … so other healthcare workers need to learn about radiation protection so we make sure we screen the request forms because there are those requests ordered by nurses and you will see that there is no need to do an X-ray so we need X-ray requests to be made by appropriate personnel which is a doctor.’ (Participant 3, HC4, M, 20–29)

‘Even if it’s clear that the patient is just having a bruise, they come with request forms having nurses signatures or no signature at all.’ (Participant 1, H3, F, 20–29)

*Sub-theme 3.5: Methods to encourage compliance:* This sub-theme examined potential strategies or methods used to enhance radiographers’ adherence and conformance with radiation protection regulations. The radiographers demonstrated an interest and a willingness to develop, and they were optimistic that this study would be instrumental in bettering their adherence to safety protocols. The radiographers highlighted potential mitigation strategies for their compliance issues. This included the education of other staff members in the PHFs surrounding radiation safety standards, as this would lower their likelihood of ordering unnecessary X-rays without proper consultations or examinations. When other employees and patients are educated, they may also be cautious around the RD, especially because some of the infrastructure is lacking:

‘… I hope this study yields good results on the issue of radiation protection in our departments.’ (Participant 3, HC4, M, 20–29)

‘… we find that we have to expose patients for unnecessary orders to rule out fractures while it’s clear there is no need … so other healthcare workers need to learn about radiation forms because there are those requests ordered by nurses and you will see that there is no need to do an X-ray so we need X-ray requests to be made by appropriate personnel which is a doctor.’ (Participant 3, HC4, M, 20–29)

‘Educate our patients because if they were to be educated about radiation protection because most of them do not know anything about radiation protection whether you protect them from ionising radiation or not, they don’t know, for them, they come for whatever they are expecting whatever they are expecting and go out.’ (Participant 7, H2, F, 30–49)

‘We are willing and ready to help with public awareness of radiation and its dangers but we need support from the MOH.’ (Participant 2, H3, M, 30–49)

Participants believed that the obstacles they face are a result of the lack of a governing body for radiographers in Eswatini, alongside the absence of a radiation protection advisory and/or authority board. They also stated that they do not know to whom to direct requests as many people in leadership are still unaware of radiography as a practice. Therefore, having a board would aid in the field’s representation at a national level or within the MOH:

‘… we need a radiation board so we will know where to send complaints and requests pertaining to radiation protection.’ (Participant 13, H6, F, 30–49)

‘… provide radiation protection officers to come now and then and see the challenges we face and work on it, that includes the equipment.’ (Participant 1, H3, F, 20–29)

‘My conclusion is that we need a radiation protection authority board.’ (Participant 10, H5, M, 50–59)

‘We are not sure of the equipment situation in as far as radiation protection is concerned.’ (Participant 8, HC3, F, 30–49)

‘… we need radiation protection officers such people are needed so that you can improve or see if there are any discrepancies on the equipment and with us radiation workers.’ (Participant 3, HC4, M, 20–29)

‘Okay, I would say that it must begin with us as radiographers. Before pushing it and blaming it on someone else, we are the ones who must engage others and give them a sense of what we are talking about. We are also the ones who must create a proper structure for our partners and let them know what we are talking about when discussing a radiology department. Therefore, moving on to the next individual, we must also engage the government. The MOH must go out and learn about the different patterns and find a way to get feedback on what is happening in the hospital departments as long as it deals with health services, and it must open an office where we can submit our concerns or complaints.’ (Participant 2, H3, M, 30–49)

## Discussions

The study aimed to explore compliance with radiation protection among radiographers in Eswatini PHFs using the TPB. This theory posits that individual behaviour is driven by behavioural intentions, which are a function of three main determinants, namely an individual’s attitude towards behaviour, subjective norms and perceived behavioural control (Nioi et al. 2018:3). The three determinants will be discussed in the following order: attitude towards radiation protection, subjective norm and perceived behavioural control.

The results of this study demonstrated favourable attitude towards radiation protection among the radiographers because they had knowledge and awareness of radiation safety, which was consistent with Dlamini and Kekana (2021:1). Their understanding was further in line with South African Radiation Control (2016:11–17).

Participants further indicated that they mainly use the lowest exposure to obtain a diagnostic quality image using lead devices, they try to avoid repeated exposure, use high kVp techniques, collimate to the area of interest and screen request forms because unauthorised staff can request unnecessary X-ray orders. However, while they showed knowledge and awareness, unfavourable attitudes were also noticed because there was conditional and limited use or application of radiation protection resulting in compliance being a personal choice. This conditional compliance often related to the focus on paediatric patients and pregnant women, which was insufficient. All patient populations, without any exception, need to be protected from unnecessary radiation exposure. This pattern of results was consistent with Lewis et al.’s (2022a:390) study conducted in South Africa; it indicated that radiographers’ knowledge of radiation protection aligned with legislated guidelines but many believed that radiation protection was a waste of time and a nuisance. The current study also concurred with Eze et al.’s (2013:1) findings, which revealed that radiographers’ knowledge of radiation safety standards was high but their adherence to radiation protection practices was low in all of the investigated institutions.

Normative beliefs are beliefs underlying subjective norms. Normative beliefs are radiographers’ belief that patients, patients’ families, coworkers, radiology managers and radiologists think they should or should not practice protection (Lewis et al. 2022b:52). According to results of this investigation, radiation safety awareness was particularly poor in patients (Dehghani 2015:116). The limited knowledge was observed from the participants responses indicating that patients’ complain that lead aprons are heavy and they never ask for it to be used when having radiographic examinations. These authors showed that, despite growing concerns regarding medical radiation exposure, there was still limited and inferior awareness of radiation-induced cancer risks among patients. Therefore, given the low patient awareness about imaging dose, it was recommended to prepare and give all patients brochures that explain safety procedures and common concerns. Information posters must be displayed in the imaging department and throughout the hospital. Moreover, hospital management should design programmes that would emphasise patient education, such as introductory talks every morning before work begins (Kamara, Okoye & Omubo-Pepple 2013:87; Naderi et al. 2021:2). Thus, organising public awareness programmes on radiation protection can also be a turning point in improving patients’ knowledge of radiation-induced effects, based on the sentiments of the participants. This initiative could yield positive results, provided that the requirements for radiation workers to comply with radiation safety standards are also met effectively.

Perceived behavioural control is based on control beliefs that account for perceived constraints and an individual’s confidence in performing the action (Lewis et al. 2022b:52). Even though some of the participants in this study indicated that using radiation protection was under their control meaning it’s up to them either they comply or not because there is no bounding policy or obligation to comply with radiation protection, some believed that radiation protection was out of their control in certain instances.

The radiographer’s responses confirmed that there is a resource challenge in their RDs, such as the different lead protective devices that play specific roles (e.g., very few participants indicated that they had thyroid shields, wrap-arounds, goggles and gloves). Participants did not have every type of lead shielding device necessary to protect the patients’ safety. They were forced to ‘get creative’ with lead aprons, which often left the devices being damaged (cracked). These findings are consistent with Bwanga and Chanda (2020:1) study conducted in Zambia, which revealed limited personal radiation protective equipment. In this study, participants specifically mentioned the aprons’ poor conditions, with repeated requests for replacements to be made. These findings were consistent with Kellens et al. (2022:2) and Dlamini and Kekana (2021:1); both studies mention a lack of legislative control in PHFs. A similar study conducted in Iran by Salmanvandi et al. (2015:1) showed that some personal shields have defects (tears, holes and cracks), and 13% of them were not acceptable in terms of ELT and needed to be replaced to better comply with radiation protection. In addition, a South African study by Lewis et al. (2022b:49) revealed that perceived behavioural control indirectly through agreement indicated the overall universal agreement that practising radiation protection would be easier if some elements or factors were available, such as the availability of lead rubber shields, recognition in the form of awards for compliance and working in a department that promotes a safety culture.

The findings of the study further revealed that the participants did not only have resource problems but faced several obstacles that hindered their ability to comply with radiation safety standards. Participants were demotivated from complying with the radiation protection because of factors related to an overload of patients; the queue(s) were too long to spend time focussing on radiation safety other than pushing the line, which was consistent with Lewis et al.’s (2022b:47) study. Patients also complained that the aprons are heavy; therefore, this made them do as the patient wants because, at the end of the day, they are not obliged to always comply with radiation safety and their colleagues did the same that became the culture or norm, which was also found by Lewis et al. (2022a:387).

Furthermore, the poor infrastructure of the RDs because of improper buildings, structure and location were a cause for concern. The walls were not lined with lead and some of the RDs were located between busy departments (such as the laboratory), causing the staff or patients in other rooms to be exposed. Moreover, one of the RD only had a curtain instead of a door, highlighting the poor structure of the department. This finding is consistent with Tombo et al. (2023:5), who demonstrated that basic infrastructure is often lacking.

Moreover, there was a tendency for unauthorised staff and/or nurses to make unnecessary X-ray requests for patients. Currently, there is no legislation preventing excessive X-ray requests without proper examinations. In some countries, X-ray requisition forms are required but can be circumvented by nursing staff in accident and emergency departments who have completed a limb assessment training programme (Auckland District Health Board 2020:2). Among other competencies, nurses must demonstrate knowledge of contraindications for requesting X-rays and the risk of unnecessary radiation exposure. The practice of nurses requesting X-rays has been pioneered in accidents and emergency departments but is now commonplace in many departments. This policy would, therefore, prevent nurses and non-medical practitioners from requesting X-rays without proper examinations to ensure prompt diagnosis and treatment, to deliver patient-focussed, high-quality care. The IAEA (n.d.b.: par. 1 & 12) recommends that any radiological procedure on an asymptomatic individual that is intended to be performed for the early detection of disease, but not as part of an approved health screening programme, shall require specific justification for that individual by the radiological medical practitioner and the referring medical practitioner, in accordance with the guidelines of relevant professional bodies or the health authority. As part of this process, the patient shall be informed in advance of the expected benefits, risks and limitations of the radiological procedure. The goal of justification is to avoid unnecessary radiological procedure, which would result in patient being unnecessarily exposed to ionising radiation and its potential risks. This study found that radiographers would benefit from nurses and/or unauthorised staff being trained to avoid unnecessary X-rays by making use of a request form. These X-ray request forms are clinical and legal documents completed by a referring clinician or their surrogate to communicate the required procedure and the reasons for the procedure (Jimah 2021:1).

Furthermore, this study found that radiographers see a need for the introduction of a national radiation regulatory body. This was consistent with Dlamini and Kekana’s (2021:1) findings. The benefits of having a regulatory body include inspection and enforcement to ensure that facilities, equipment and work performance meet all requirements (IAEA 2013:17). The participants added that this lack of regulatory body furthers the issue of radiation safety non-compliance. A government is required to establish a national regulatory body to regulate the introduction and conduct of any practice involving sources of radiation (IAEA 2004:5). For this problem to be resolved, the government must be fully engaged. This was also confirmed in a study conducted by Maina, Motto and Hazell (2020:1). wherein they indicated that there was a need for concerted efforts between the Rwanda Utilities Regulatory Authority, the MOH (government), the University of Rwanda and hospital management to improve the radiation safety culture.

This study’s participants believed that once the government introduces a regulatory body, it would naturally lead to the introduction of Radiation Protection Officers (RPOs). The role of RPOs is to prevent unnecessary exposure (or ALARA) (Morgan & Konerth 2021:1). Furthermore, RPOs should report non-compliance with safety standards and provide what is needed to ensure compliance (Morgan & Konerth 2021:4). This could explain why compliance is so low, as RPOs are non-existent. The findings of this study further indicated that participants wished they had RPOs who could inspect their departments periodically, thus ensuring that radiography essentials were available and providing a contact point if such equipment is lacking. The need to monitor RDs is consistent with finding of Kamara et al. (2013:87).

In addition, Lakhwani et al. (2019:742) emphasised that regular surveillance of radiation exposure protection should be part of RPOs’ duties. This is however, not the case in Eswatini. This is also consistent with Zekioğlu and Parlar (2020:1), who showed that legislation in Türkiye indicated that a Radiation Safety Committee must be established in hospitals. These committees have duties such as providing radiation safety, regular hospital training and preparing a radiation safety handbook, all of which do not exist in Eswatini PHFs.

### Limitations

While this study achieved what it set out to, there were some limitations involved in the data collection process. The researcher received no funding, which delayed the process; data were collected through one-on-one, in-person interviews and participants were in locations distant from one another, therefore the cost of travelling to and from PHFs was challenging. Data were collected as initially hoped; however, the timeline was longer than first anticipated.

Qualitative research is restricted to a particular phenomenon in a certain population and within a specific context. Hence, generalisability is not an expected attribute (Leung 2015:326). Therefore, the study’s findings cannot be generalised to private sector populations. A qualitative approach was used in this study through one-on-one interviews as the primary data collection instrument. Although these interviews were in-depth, a mixed-method approach would have been beneficial. Creswell and Plano Clark (2011:12) assert that mixed-methods can compensate for weaknesses associated with a study’s quantitative or qualitative design. The mixed-methods design allows the researcher to collect data with any tool, rather than being limited to only one of the tools associated with either quantitative or qualitative research (Creswell & Plano Clark 2011:12).

### Recommendations

Based on the results, the researcher recommends that the government, through the MOH, should:

- Establish, through legislation, a national independent regulatory body to regulate the introduction and conduct of any practice involving radiation sources.
- Provide RPOs to hospitals to ensure high radiation safety standards for patients.
- Inspect the lead protective devices.
- Train and develop the RPOs.
- Assist in evaluating RD buildings.
- Alongside RD heads of departments as primary influencers, assist in enforcing the staff’s compliance with radiation protection and lead by example.

Future research may also concentrate on assessing the implementation of this study’s recommendations. The research could investigate the recommendations’ effectiveness in improving compliance with radiation safety standards.

## Conclusion

Participants showed both unfavourable/negative and favourable/positive attitudes to radiation safety standards. They showed a positive attitude and a good knowledge of radiation protection. Despite this, compliance with radiation protection focussed more on paediatric patients and pregnant women. This subjectivity was the paramount negative attitude found in this study; this attitude further contributed to observing or considering subjective norms linked to their routine behaviour because the routine behaviour that developed prevailed and became a departmental culture among the participants. The PHFs’ culture must promote compliance with radiation protection fully. This study’s findings strongly imply that action is needed to discourage or stop this culture from influencing future radiographers.

Furthermore, perceived behavioural control in the findings showed that in as much as the participants wanted to comply, they were discouraged by their experiences, which is reflected in the resources that the individual has to achieve the behaviour (Li et al. 2023:4). In Eswatini, compliance with radiation safety standards was also influenced by improper buildings, structure and location. The lack of motivation, encouragement or enforcement from seniors also contributed to non-compliance. Motalebi et al. (2021:5) indicated that gaining the support of influential people in the workplace is an effective intervention to ensure compliance. Moreover, perceived behavioural control showed that the above-mentioned factors/limitations and experiences reduced optimal compliance with radiation protection. The radiographers were not obliged to comply because no radiation regulatory body, policy or RPOs is available in Eswatini; this has resulted in compliance being a personal choice. Nevertheless, the participants suggested ways to promote compliance, specifically the introduction of a radiation authority board in the country.
